# Serum Anion Gap Predicts All-Cause Mortality in Patients with Advanced Chronic Kidney Disease: A Retrospective Analysis of a Randomized Controlled Study

**DOI:** 10.1371/journal.pone.0156381

**Published:** 2016-06-01

**Authors:** Sung Woo Lee, Sejoong Kim, Ki Young Na, Ran-hui Cha, Shin Wook Kang, Cheol Whee Park, Dae Ryong Cha, Sung Gyun Kim, Sun Ae Yoon, Sang Youb Han, Jung Hwan Park, Jae Hyun Chang, Chun Soo Lim, Yon Su Kim

**Affiliations:** 1 Department of Internal Medicine, Eulji General Hospital, Seoul, 01830, Korea; 2 Department of Internal Medicine, Seoul National University Bundang Hospital, Seongnam, 13620, Korea; 3 Department of Internal Medicine, Seoul National University College of Medicine, Seoul, 03080, Korea; 4 Department of Internal Medicine, National Medical Center, Seoul, 04564, Korea; 5 Department of Internal Medicine, Yonsei University College of Medicine 03722, Seoul, Korea; 6 Department of Internal Medicine, Seoul St. Mary's Hospital, The Catholic University of Korea, Seoul, 06591, Korea; 7 Department of Internal Medicine, Korea University Ansan-Hospital, Korea University, Ansan, Gyeonggi-do, 15355, Korea; 8 Department of Internal Medicine, Hallym University Sacred Heart Hospital, Anyang, Gyeonggi-do, 14068, Korea; 9 Department of Internal Medicine, Uijeongbu St. Mary's Hospital, The Catholic University of Korea, Uijeongbu, Gyeonggi-do, 11765, Korea; 10 Department of Internal Medicine, Inje University Ilsan-Paik Hospital, Goyang, Gyeonggi-do, 10380, Korea; 11 Department of Internal Medicine, Konkuk University School of Medicine, Seoul, 05030, Korea; 12 Department of Internal Medicine, Gachon University Gil Medical Center, Gachon University of Medicine and Science, Incheon, 21565, Korea; 13 Department of Internal Medicine, Seoul National University Boramae Medical Center, Seoul, 08708, Korea; 14 Kidney Research Institute, Seoul National University, Seoul, 03080, Korea; Ichan School of Medicine at Mount Sinai, UNITED STATES

## Abstract

**Background and Objectives:**

Cardiovascular outcomes and mortality rates are poor in advanced chronic kidney disease (CKD) patients. Novel risk factors related to clinical outcomes should be identified.

**Methods:**

A retrospective analysis of data from a randomized controlled study was performed in 440 CKD patients aged > 18 years, with estimated glomerular filtration rate 15–60 mL/min/1.73m^2^. Clinical data were available, and the albumin-adjusted serum anion gap (A-SAG) could be calculated. The outcome analyzed was all-cause mortality.

**Results:**

Of 440 participants, the median (interquartile range, IQR) follow-up duration was 5.1 (3.0–5.5) years. During the follow-up duration, 29 participants died (all-cause mortality 6.6%). The area under the receiver operating characteristic curve of A-SAG for all-cause mortality was 0.616 (95% CI 0.520–0.712, *P* = 0.037). The best threshold of A-SAG for all-cause mortality was 9.48 mmol/L, with sensitivity 0.793 and specificity 0.431. After adjusting for confounders, A-SAG above 9.48 mmol/L was independently associated with increased risk of all-cause mortality, with hazard ratio 2.968 (95% CI 1.143–7.708, *P* = 0.025). In our study, serum levels of beta-2 microglobulin and blood urea nitrogen (BUN) were positively associated with A-SAG.

**Conclusions:**

A-SAG is an independent risk factor for all-cause mortality in advanced CKD patients. The positive correlation between A-SAG and serum beta-2 microglobulin or BUN might be a potential reason. Future study is needed.

**Trial Registration:**

Clinicaltrials.gov NCT 00860431

## Introduction

The prevalence of chronic kidney disease (CKD) ranges between approximately 7% - 10% in the general population [1]. CKD is dangerous because it is independently associated with mortality even after adjusting for various chronic conditions such as hypertension, diabetes and major adverse cardiac events [2, 3]. Therefore, it is of utmost importance to identify biomarkers predicting poor outcomes in CKD. Several candidates have been proposed [4], but, these biomarkers are not always applicable in routine practice. Thus, novel and easily accessible biomarkers should be identified.

The serum anion gap (SAG) is easily calculated from the electrolytes. Along with differential diagnosis of acid-base disorders, it can be used to assess laboratory quality control and diagnose intoxications and paraproteinemias [5]. However, recent studies have suggested the expanded use of SAG as a biomarker. Farwell and colleagues analyzed the data from the National Health and Nutrition Examination Survey (NHANES). They proposed that increased SAG is associated with blood pressure, insulin resistance, and inflammation in the healthy general population [6–8]. Moreover, SAG is an independent risk factor for mortality in acute myocardial infarction [9], critical illness [10], and an otherwise healthy elderly population [11].

SAG is elevated and related to mortality even in early kidney disease [12], and high SAG is associated with CKD progression [13]. Therefore, we hypothesized that SAG might predict mortality in advanced CKD patients. We performed the current study to identify whether SAG was associated with all-cause mortality using data from a prospective randomized controlled study, KSTAR (Kremezin STudy Against Renal disease progression in Korea).

## Material and Methods

### Study population

**Fig 1:**
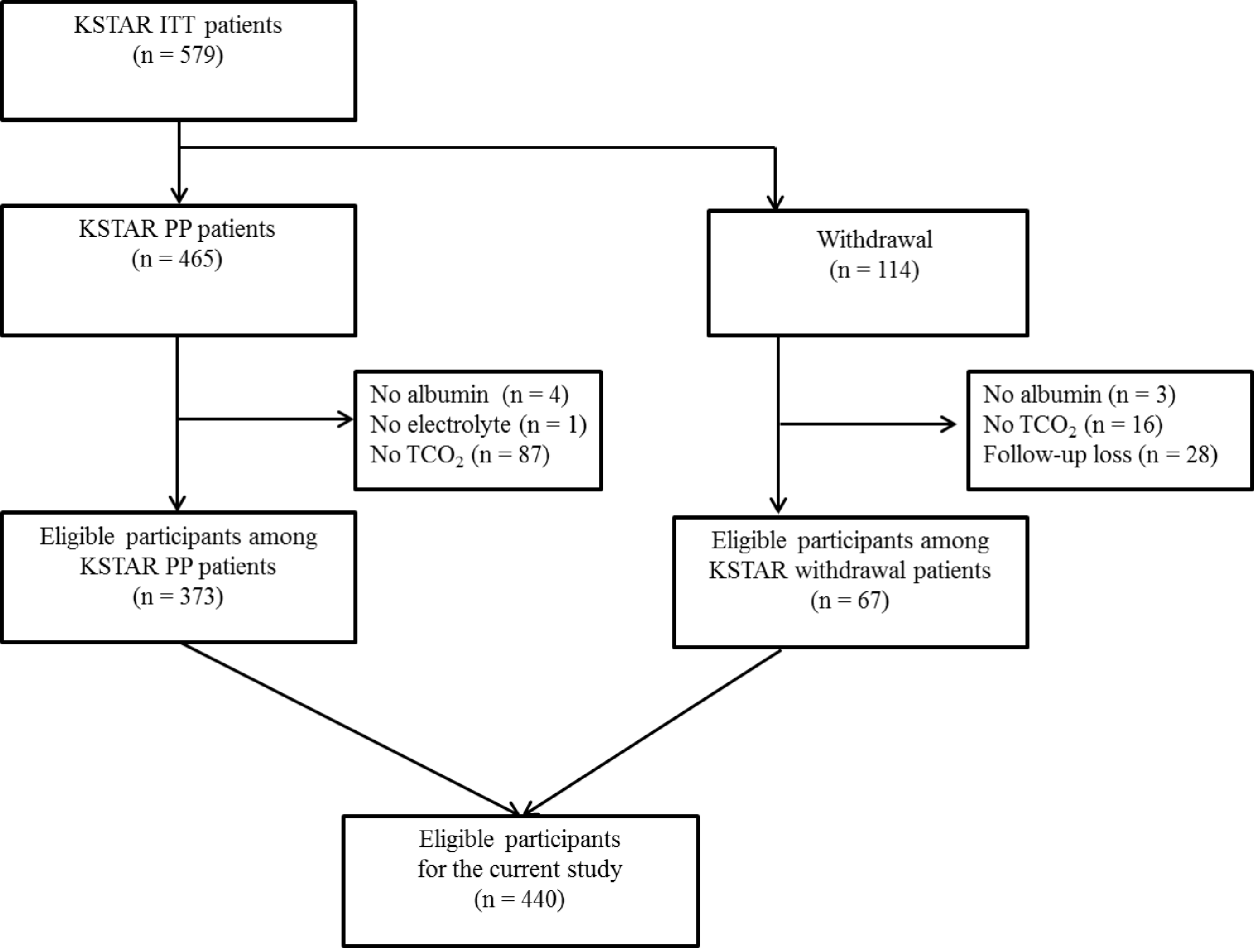
Patient selection algorithm. ITT, intention to treat; PP, per protocol.

KSTAR was a prospective, 11-center, randomized, open–label, controlled study (Clinicaltrials.gov: NCT 00860431). The detailed study protocol is described elsewhere [14]. In brief, participants with advanced CKD recruited from March 2009 to August 2010 were followed up for 36 months. Those aged ≥ 18 years, with estimated glomerular filtration rate (eGFR) by the Cockcroft-Gault equation of 15–59 mL/min/1.73 m^2^, and who had a measured or expected decline of kidney function of ≥ 2.5 mL/min/1.73 m^2^ for six months or ≥ 5 mL/min/1.73 m^2^ for 12 months, were included in the original study. The study protocol was approved by the institutional review boards of the participating centers, and the study was performed in compliance with the principles of the Declaration of Helsinki. In addition to the original data, we retrospectively collected data on the status of survival as of July 2015 through review of medical records. The retrospective expansion of the follow-up duration was also approved by the institutional review boards (IRBs) of the participating centers: Seoul National University Bundang Hospital (B-0812/066-006), Seoul National University Bundang Hospital (0810-009-259), Seoul National University Boramae Medical Center (06-2009-7), Yonsei University Hospital (4-2008-0586), Korea University Ansan-Hospital(AS0884), Hallym University Sacred Heart Hospital (2009-I004), Inje University Ilsan-Paik Hospital (IB-0811-076), Konkuk University Hospital (KUH1010125), and Gachon University Gil Medical Center (GIRBA2010). The need for informed consent for the retrospective expansion was waived by the IRBs of participating hospitals because the study did not infringe on patient privacy or health status. Patient records and information were anonymized and de-identified prior to analysis. Of 579 participants, 440 were eligible for the analysis (Fig 1).

### Definition and measurements

The eGFR was calculated by the Chronic Kidney Disease EPIdemiology collaboration equation [15]. The SAG was defined using the following equation: SAG (initial) = serum sodium [mEq/L] − (serum chloride [mEq/L] + serum total CO_2_ [mEq/L]). Since approximately 80% of SAG is due to the sum of anionic charges on blood proteins, and albumin is the most abundant, hypoalbuminemia could falsely lower the SAG [5]. Thus SAG needed to be adjusted for serum albumin using the following equation: albumin-adjusted SAG (A-SAG) = SAG (initial) + 2.5 × (4 –serum albumin [g/dL]) [16]. Serum total indoxyl sulfate (IS) was measured by a high performance liquid chromatography fluorescence detector (Agilent 1100 series, USA).

### Statistical analysis

**Fig 2:**
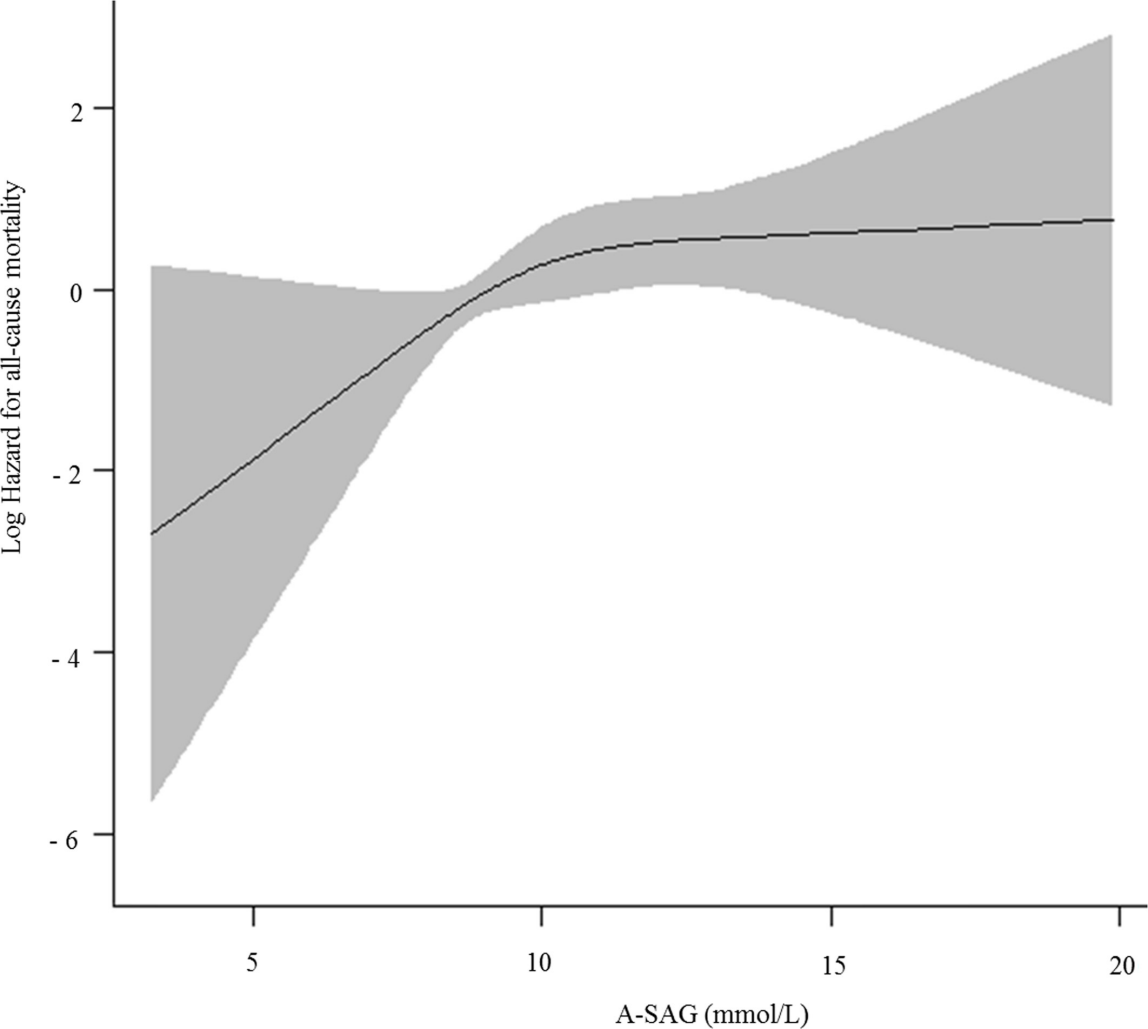
Restricted cubic spline curve of albumin-adjusted serum anion gap (A-SAG) for all-cause mortality. As A-SAG increased, the log hazard of all-cause mortality increased, but nonlinearly.

Values were expressed as mean ± standard deviation (SD) for continuous variables and percentage for categorical variables. Differences were evaluated by *Student`s t*-test for continuous variables and the chi-square test for categorical variables. Normality was assessed using a Q-Q plot. If continuous variables were not normally distributed, values were expressed as median (interquartile range, IQR), and the difference was evaluated by the Mann-Whitney *U* test. The survival curve was evaluated by the Kaplan-Meier method. To adjust for confounding effects among variables, we performed Cox-proportional hazard regression analysis. The assumption of proportional hazard was verified by a log minus log plot. The integrated variables were known risk factors or variables with *P* < 0.05 in univariate analysis. For linearity assessment between continuous variables and all-cause mortality, we fitted restricted cubic splines using R Version 3.03 (R Core Team, 2014) [17] with “rms” package [18]. Since the restricted cubic spline curve (Fig 2) and multivariate Cox-proportional hazard regression of A-SAG quintile (S1 Table) revealed a non-linear association between A-SAG and all-cause mortality, all participants were divided into two groups of ≥ vs. < the threshold of A-SAG calculated from the area under the receiver operating characteristic curve (AUROC) using R Version 3.03 (R Core Team, 2014) with “pROC” package [19]. The best threshold was calculated by obtaining the best Youden index (sensitivity + specificity—1). For comparison of mean and linear regression of serum total IS, logarithmic transformation was performed, since it was non-normally distributed. *P* < 0.05 was considered to have statistical significance. All analysis unless otherwise specified was performed using SPSS Version 22 (IBM Corp. released 2013, Armonk, NY: IBM Corp).

## Results

Of 440 participants, the median (IQR) follow-up duration was 5.1 (3.0–5.5) years. At baseline, mean age was 56.8 ± 13.1 years and men were 294/440 (66.8%). The mean eGFR was 23.2 ± 6.8 mL/min/1.73 m^2^ and median urine protein creatinine ratio (UPCR) (IQR) was 1.2 (0.4–2.9) g/g. The proportion of diabetic nephropathy as a cause of CKD was 204/440 (46.4%). During the follow-up duration, 29 participants died (all-cause mortality 6.6%).

**Fig 3:**
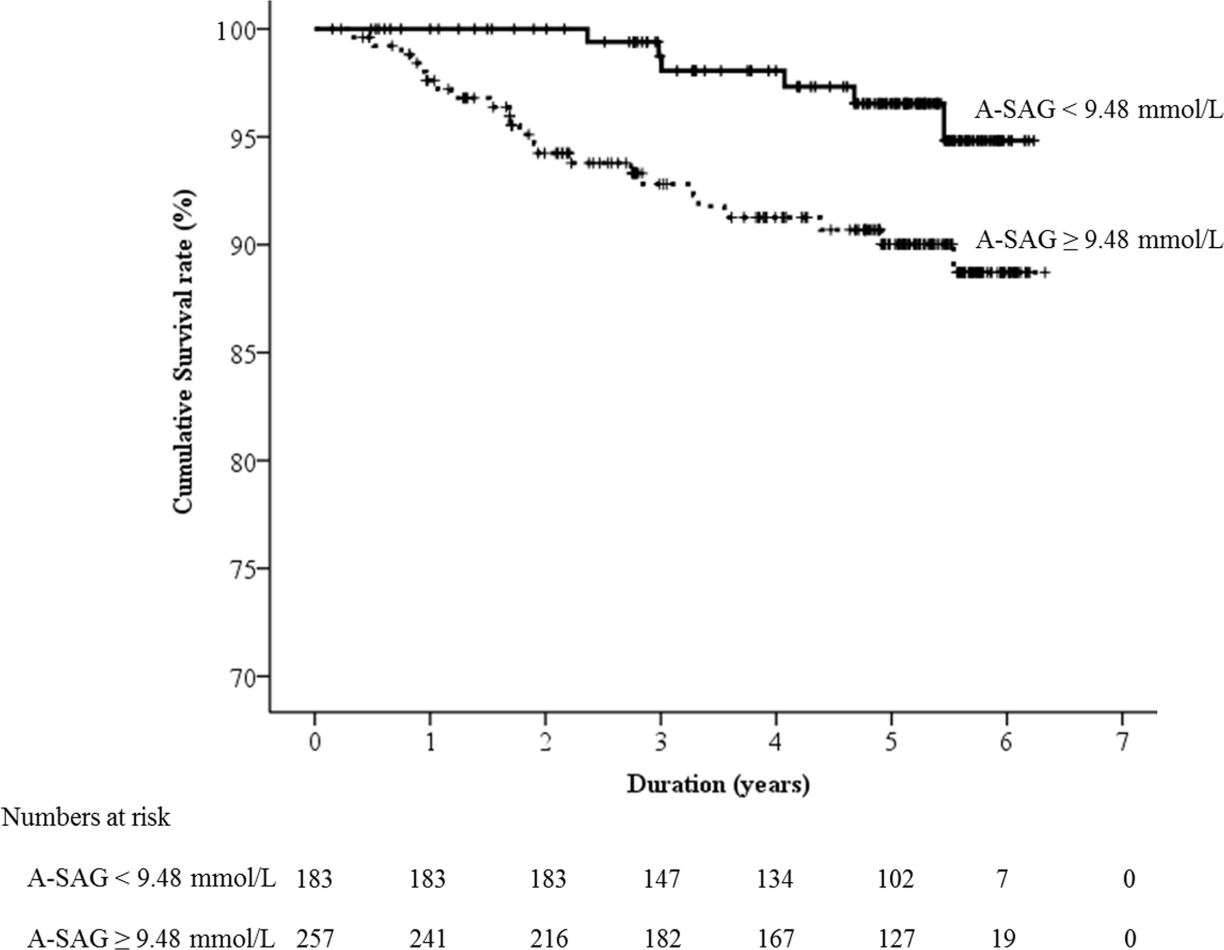
Kaplan-Meier survival curve according to the albumin-adjusted serum anion gap (A-SAG) status. Mean (95% CI) survivals of < and ≥ 9.48 mmol/L of A-SAG groups were 6.1 (6.0–6.2) years and 5.9 (5.7–6.1) years, respectively.

We evaluated several cut-points of A-SAG and found only 9.3 mmol/L, the cut-point between the 2^nd^ and 3^rd^ quintile was significantly associated with all-cause mortality (S2 Table). In discrimination analysis, the AUROC of A-SAG for the all-cause mortality was 0.616 (95% confidence interval [CI] 0.520–0.712, *P* = 0.037). The best threshold of A-SAG for all-cause mortality was 9.48 mmol/L, with sensitivity 0.793 and specificity 0.431. We compared the baseline characteristics of the participants according to the status of A-SAG threshold (Table 1). The means of A-SAG in ≥ and < 9.48 mmol/L of A-SAG groups were 11.5 ± 1.9 mmol/L and 7.8 ± 1.4 mmol/L, respectively. The A-SAG ≥ 9.48 mmol/L group showed higher male proportion than the A-SAG < 9.48 mmol/L group. Although serum level of inorganic phosphorus was not different between the two A-SAG groups, the serum levels of potassium and chloride were significantly lower in A-SAG ≥ 9.48 mmol/L group than A-SAG < 9.48 mmol/L group. Participants with A-SAG ≥ 9.48 mmol/L showed lower eGFR and higher blood urea nitrogen (BUN) than those with A-SAG < 9.48 mmol/L. The all-cause mortality of ≥ and < 9.48 mmol/L of A-SAG groups was 23/257 (8.9%) and 6/183 (3.3%), respectively (*P* = 0.019). In Kaplan-Meier survival curve analysis (Fig 3), the A-SAG ≥ 9.48 mmol/L group showed poorer patient survival than the A-SAG < 9.48 mmol/L group.

**Table 1:** Baseline characteristics according to the status of A-SAG threshold.

	A-SAG < 9.48 mmol/L (n = 183)	A-SAG ≥ 9.48 mmol/L (n = 257)	*P*
Age (years)	56.0 ± 13.1	57.3 ± 13.2	0.297
Male sex	132/183 (72.1)	162/257 (63.0)	0.046
Diabetic nephropathy	83/183 (45.4)	121/257 (47.1)	0.720
Systolic BP (mmHg)^b^	131.0 ± 15.0	130.9 ± 14.1	0.931
eGFR (mL/min/1.73m^2^)	24.2 ± 6.8	22.5 ± 6.7	0.012
Blood urea nitrogen (mmol/L)	14.3 ± 4.1	15.9 ± 5.6	<0.001
Creatinine (μmol/L)^a^	229.8 (203.3–282.9)	238.7 (203.3–291.7)	0.157
Urine protein creatinine ratio (g/g)^a^^,^^b^	1.1 (0.4–2.9)	1.3 (0.5–2.9)	0.315
Total calcium (mmol/L)^b^	2.2 ± 0.1	2.2 ± 0.2	0.466
Inorganic phosphorus (mmol/L)	1.2 ± 0.2	1.3 ± 0.3	0.087
Serum glucose (mmol/L)^b^	6.3 ± 2.2	6.6 ± 2.8	0.287
Serum albumin (g/L)^a^	41.0 (39.0–44.0)	40.0 (38.0–43.0)	0.081
C-reactive protein (nmol/L)^a^^,^^b^	10.5 (1.9–37.9)	12.4 (1.0–58.6)	0.351
Sodium (mmol/L)	140.6 ± 2.2	140.5 ± 2.4	0.705
Potassium (mmol/L)	5.1 ± 0.6	5.0 ± 0.6	0.045
Chloride (mmol/L)	108.7 ± 3.4	107.5 ± 3.8	0.001
Total CO_2_ (mmol/L)	24.0 ± 2.9	21.6 ± 3.3	< 0.001
SAG (mmol/L)	7.9 ± 1.7	11.5 ± 2.1	< 0.001
A-SAG (mmol/L)	7.8 ± 1.4	11.5 ± 1.9	< 0.001
Log serum total IS (mg/L)^b^	1.4 ± 1.1	1.4 ± 1.3	0.970
Serum B2MG (mg/L)^b^	6.5 ± 2.5	6.9 ± 2.7	0.102

Values were expressed as mean ± standard deviation for continuous variables and n/total (%) for categorical variables. Differences were evaluated by t-test or Mann-Whitney U test for continuous variables, and chi-square test for categorical variables. BP, blood pressure; eGFR, estimated glomerular filtration rate; SAG, serum anion gap; A-SAG, albumin-adjusted serum anion gap; IS, indoxyl sulfate; B2MG, beta-2 microglobulin.

^a^ denotes variables with non-normal distribution. Values were expressed as median (interquartile range) and differences were evaluated by Mann-Whitney U test in these variables.

^b^ denotes incomplete data. The missing rates of the variables were less than 6.1%.

We performed univariate and multivariate Cox-proportional hazard regression analysis to identify factors associated with all-cause mortality (Table 2). The A-SAG ≥ 9.48 mmol/L was significantly associated with increased risk of all-cause mortality. In addition, older age, lower eGFR, higher serum beta-2 microglobulin (B2MG) and BUN were also significantly associated with increased risk of all-cause mortality. After adjusting for confounders, A-SAG ≥ 9.48 mmol/L was still associated with the increased risk of all-cause mortality, with hazard ratio 2.968 (95% CI 1.143–7.708, *P* = 0.025).

**Table 2:** Factors associated with all-cause mortality in Cox-proportional hazard regression analysis.

	Univariate		Multivariate	
	HR (95% CI)	*P*	HR (95% CI)	*P*
Age (per 1 year increase)	1.099 (1.055–1.144)	< 0.001	1.086 (1.044–1.130)	< 0.001
Sex (male vs. female)	1.381 (0.612–3.118)	0.437	2.251 (0.902–5.613)	0.082
Diabetic nephropathy (yes vs. no)	1.713 (0.818–3.588)	0.154	-	-
Systolic BP (per 1 mmHg increase)	1.013 (0.987–1.039)	0.331	-	-
Randomization (intervention vs. control)	0.971 (0.469–2.012)	0.937	-	-
eGFR (per 1 mL/min/1.73m^2^ increase)	0.899 (0.842–0.960)	0.002	0.933 (0.832–1.046)	0.235
SAG (per 1 mmol/L increase)	1.096 (0.959–1.253)	0.179	-	-
A-SAG (≥ vs. < 9.48 mmol/L)	2.917 (1.187–7.166)	0.020	2.968 (1.143–7.708)	0.025
Inorganic phosphorus (per 1mmol/L increase)	1.049 (0.227–4.840)	0.951	0.722 (0.119–4.373)	0.723
Serum Total CO_2_ (per 1 mmol/L increase)	0.937 (0.843–1.041)	0.227	1.036 (0.919–1.167)	0.567
Serum B2MG (per 1mg/L increase)	1.238 (1.107–1.383)	<0.001	1.157 (0.962–1.390)	0.121
Blood urea nitrogen (per mmol/L increase)	1.077 (1.015–1.143)	0.015	1.006 (0.916–1.105)	0.897
Log serum total IS (per mg/L increase)	1.309 (0.928–1.845)	0.125	-	-

Known risk factors or variables with *P* < 0.05 were integrated in multivariate analysis. BP, blood pressure; eGFR, estimated glomerular filtration rate; SAG, serum anion gap; A-SAG, albumin-adjusted serum anion gap; B2MG, beta-2 microglobulin; IS, indoxyl sulfate.

**Fig 4:**
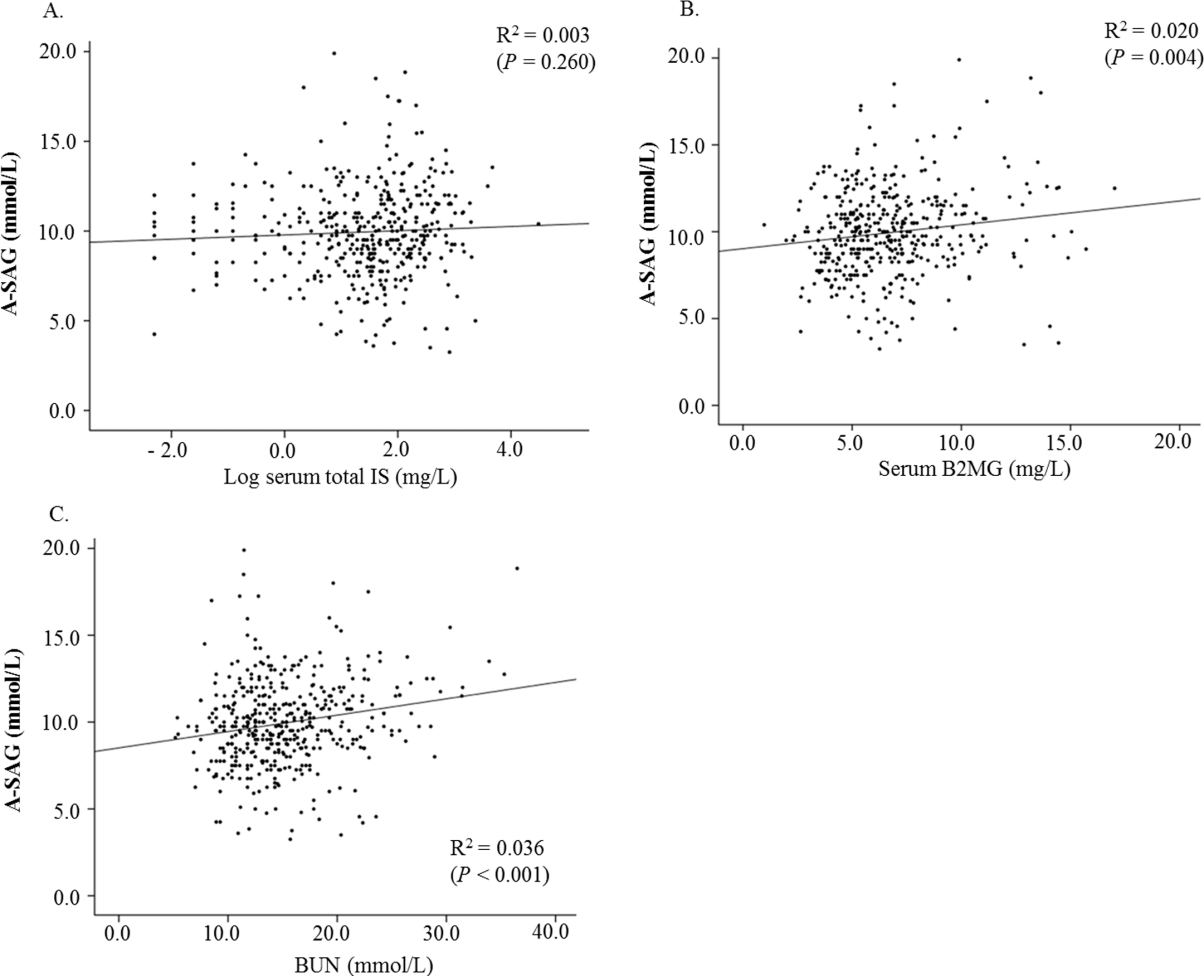
Associations between albumin-adjusted serum anion gap (A-SAG) and uremic toxins. A, B, and C designated serum total indoxyl sulfate (IS), serum beta-2 microglobulin (B2MG) and blood urea nitrogen (BUN), respectively.

To determine the reason for the association between A-SAG and all-cause mortality, we performed linear regression analysis for A-SAG and uremic toxins. The serum total IS was not associated with A-SAG (Fig 4A), with beta (95% CI) 0.118 (-0.088–0.325). However, serum B2MG (Fig 4B) and BUN (Fig 4C) were positively associated with A-SAG, with beta (95% CI) 0.137 (0.044–0.230) and 0.094 (0.049–0.140), respectively.

## Discussion

We evaluated the usefulness of A-SAG as a potential biomarker for all-cause mortality in advanced CKD patients. SAG is easily calculated from serum or plasma electrolytes. Since SAG is significantly affected by the serum albumin level [5], we used A-SAG to increase the predictive value [16]. To our knowledge, this is the first study to explore the clinical usefulness of A-SAG in advanced CKD patients. In addition, our study suggested that A-SAG ≥ 9.48 mmol/L in patients with advanced CKD might be derived from retained middle-molecular or small water-soluble uremic toxins.

In our discrimination analysis, the best threshold of A-SAG for all-cause mortality was 9.48 mmol/L, which has traditionally been considered in the normal range [5]. Patients with A-SAG ≥ 9.48 mmol/L showed 2.968 times higher risk of all-cause mortality than those with A-SAG < 9.48 mmol/L. Although high SAG is generally thought to be tightly linked with metabolic acidosis [20], our data suggested that A-SAG ≥ 9.48 mmol/L might be predictive of all-cause mortality in advanced CKD patients independently of metabolic acidosis. Abramowitz et al. reported similar results after analyzing 11,957 adults in NHANES 1999–2004 [12]. Compared to the first quartile of A-SAG, the fourth quartile was associated with increased risk of all-cause mortality, with hazard ratio 1.62 (95% CI, 1.19–2.21, *P* trend = 0.007). However, the meaning of their results differs from that of our study, since the majority (over 90%) of the study participants had normal kidney function (eGFR above 60 mL/min/1.73m^2^). In contrast, our study participants were only made up of advanced CKD patients, who a have higher risk of death [2, 3, 21] and need more care. Although Abramowitz et al. used A-SAG, the values were far lower than the usual range [5, 12]. In contrast, we adopted an equation for albumin-adjustment with a range similar to the usual reference [16], and hence more easily applicable. Togawa et al. also analyzed 42 patients with advance CKD (eGFR 7.1–52.0 mL/min/1.73vm^2^) for the association between A-SAG and CKD progression [13]. Although they suggested that A-SAG was significantly associated with delta eGFR/6 months in multiple linear regression (beta 0.45, *P* < 0.01), they did not evaluate the association with mortality.

The reason why A-SAG ≥ 9.48 mmol/L is predictive of mortality in advanced CKD patients remains unclear. Although several studies have suggested that elevated SAG is associated with increased risk of mortality in various populations [9–12], none of the previous studies provided relevant evidence. The lower eGFR in A-SAG ≥ 9.48 mmol/L group can explain some of the excess mortality. But multivariate analysis revealed that the impact of A-SAG was valid even after controlling the effect of eGFR. According to our study results, we speculate that uremia itself might be the reason why elevated AG is associated with increased risk of mortality in advanced CKD patients. Uremia is an important cause of elevated AG [20]. In retention of uremic toxins, decreased renal tubular secretion also plays an important role in addition to decreased glomerular filtration since some toxins, such as middle molecules, are too large to be filtered well [22]. There is increasing evidences that a high level of uremic toxin is associated with increased risk of mortality [23–26]. In our study, we analyzed serum levels of total IS, B2MG, and BUN, as representatives of highly protein-bound, middle-molecular and small-water soluble uremic toxins, respectively [27, 28]. Since serum B2MG has a negative charge at physiological pH, it acts as anion [29]. We assumed this was the reason why the serum level of B2MG was positively associated with the level of A-SAG. BUN was also positively related to the level of A-SAG. We also found that the serum levels of B2MG and BUN were associated with increased risk of all-cause mortality, which was consistent with the results of previous studies [23–26]. However, serum level of total IS was not associated with the level of A-SAG and all-cause mortality in the study. Serum IS has a high affinity to serum protein, mostly albumin [23]. Therefore serum level of total IS cannot reflect the serum level of free IS well. In fact, most of the literature showing a relationship between serum IS and mortality used the serum level of free IS [24]. We believed this was the reason why the serum level of total IS was not associated with the level of A-SAG and all-cause mortality. Based on our result, we infer that the increased risk of A-SAG ≥ 9.48 mmol/L for all-cause mortality might be derived from the positive relationship between A-SAG and middle-molecular or small water-soluble uremic toxins.

Although the contribution of uremic toxins in A-SAG might be an important reason for the increased risk of all-cause mortality, we also speculate that there may be different underlying mechanisms, since the explanatory power of uremic toxins for A-SAG was not high. We believe that A-SAG could a surrogate marker for a poor lifestyle. Gannon et al. explored dietary intake with respect to acid-base balance in an elderly UK population [30]. They calculated net endogenous acid production, which is closely associated with dietary acids and organic anions. In their analysis, higher dietary acidity was significantly associated with higher consumption of protein (*P* < 0.02). Since most bases of dietary acids are weak organic anions [30], elevated SAG would reflect high dietary acid and protein intake, which is harmful to CKD patients [31]. Elevated SAG is also related to poor cardiorespiratory fitness. Abramowitz et al. analyzed the data of NHANES 1999–2004 for the association between elevated SAG and cardiorespiratory fitness, as determined by submaximal exercise testing [32]. After adjustment, a 1-SD increase of SAG was associated with increased odds for poor fitness (odds ratio 1.30, 95% CI 1.15–1.48). We surmise that the poor lifestyle reflected by elevated SAG might be a potential reason why elevated SAG is associated with increased risk of all-cause mortality.

Although the serum level of inorganic phosphrous was equal in both < and ≥ 9.48 mmol/L of A-SAG groups in the current study, hyperphosphatemia can raise SAG [5]. Since hyperphosphatemia and secondary hyperparathyroidism are associated with mortality [33–35], we assumed that hyperphosphatemia and related secondary hyperparathyroidism in patients with elevated SAG are also a good explanation for the association between SAG and mortality.

The current study had several limitations. First, the current study was retrospective, and important values such as total CO_2_ were not available at randomization and subsequent visits since the original study did not aim to evaluate the impact of SAG on clinical outcomes. In the study, the outcome relied on retrospective medical chart review, which might underreport the true mortality. In addition, we did not have detailed data on causes of death, except for cardiovascular mortality, which was too low (2.5%) to perform reliable analysis. Second, the association between uremic toxins and A-SAG was analyzed cross-sectionally, and a causal relationship could not be confirmed. Despite this weakness, our results are useful, because they show the relationship between uremic toxins and A-SAG for the first time. Finally, all participants of the original study were Asians, which limits the generalizability.

In conclusion, the level of A-SAG is an independent risk factor for all-cause mortality in advanced CKD patients. Currently, nephrologists who care advanced CKD patients have no easily accessible biomarkers beyond serum creatinine. In this regard, A-SAG can be a cheap and effective guide. Based on our study results, clinicians can warn a patient with A-SAG ≥ 9.48 mmol/L for the poor prognosis, and recommend stricter adherence to the medication and good life style with more frequent clinic visits for timely interventions. The effective way to lower A-SAG and the efficacy of A-SAG lowering intervention needs further investigation.

## Supporting Information

S1 Table Multivariate Cox-proportional hazard regression of A-SAG quintile and all-cause mortality. (DOCX)

S2 Table Multivariate Cox-proportional hazard regression of several A-SAG cut-points for the all-cause mortality. (DOCX)
