# Supplementary material for: Serum Anion Gap Predicts All-Cause Mortality in Patients with Advanced Chronic Kidney Disease: A Retrospective Analysis of a Randomized Controlled Study
Source: PLoS One. 2016 Jun 1;11(6):e0156381. doi: 10.1371/journal.pone.0156381 (PMC4889106; doi:10.1371/journal.pone.0156381)
Supplement: S1 Table — (DOCX) [file pone.0156381.s001.docx]

S1 Table. Multivariate Cox-proportional hazard regression of A-SAG quintile and all-cause mortality.

| Quintile of A-SAG (mmmol/L) | Adjusted HR (95% CI) | *P* |
| --- | --- | --- |
| 2Q (8.2 – 9.3) vs. 1Q (< 8.2) | 2.305 (0.414 - 12.825) | 0.340 |
| 3Q (9.3 – 10.4) vs. 1Q (< 8.2) | 4.844 (0.952 - 24.651) | 0.057 |
| 4Q (10.4 – 11.8) vs. 1Q (< 8.2) | 4.119 (0.786 - 21.587) | 0.094 |
| 5Q (≥ 11.8) vs. 1Q (< 8.2) | 6.283 (1.139 - 34.672) | 0.035 |

Adjusting variables were age, sex, eGFR, B2MG, BUN, phosphorus, potassium, and total CO_2._
