# Supplementary material for: Serum Anion Gap Predicts All-Cause Mortality in Patients with Advanced Chronic Kidney Disease: A Retrospective Analysis of a Randomized Controlled Study
Source: PLoS One. 2016 Jun 1;11(6):e0156381. doi: 10.1371/journal.pone.0156381 (PMC4889106; doi:10.1371/journal.pone.0156381)
Supplement: S2 Table — (DOCX) [file pone.0156381.s002.docx]

S2 Table. Multivariate Cox-proportional hazard regression of several A-SAG cut-points for the all-cause mortality.

| Cut-points of A-SAG (mmol/L) | Adjusted HR (95% CI) | *P* |
| --- | --- | --- |
| 8.0 (≥ vs. <) | 3.329 (0.753 - 14.722) | 0.113 |
| 8.2 (≥ vs. <)* | 3.728 (0.837 - 16.598) | 0.084 |
| 9.0 (≥ vs. <) | 2.097 (0.777 - 5.661) | 0.144 |
| 9.3 (≥ vs. <)* | 2.954 (1.094 - 7.976) | 0.033 |
| 10.0 (≥ vs. <) | 1.861 (0.780 - 4.437) | 0.161 |
| 10.4 (≥ vs. <)* | 1.785 (0.768 - 4.151) | 0.178 |
| 11.0 (≥ vs. <) | 1.808 (0.734 - 4.456) | 0.198 |
| 11.8 (≥ vs. <)* | 1.814 (0.710 - 4.637) | 0.214 |

Adjusting variables were age, sex, eGFR, B2MG, BUN, phosphorus, potassium, and total CO_2._

* Cut-points for the quintile of A-SAG.
